# Tunable Dual-Broadband Terahertz Absorber with Vanadium Dioxide Metamaterial

**DOI:** 10.3390/nano12101731

**Published:** 2022-05-18

**Authors:** Hengli Feng, Zuoxin Zhang, Jingyu Zhang, Dongchao Fang, Jincheng Wang, Chang Liu, Tong Wu, Guan Wang, Lehui Wang, Lingling Ran, Yang Gao

**Affiliations:** 1 School of Electronic Engineering, Heilongjiang University, Harbin 150080, China; 2201622@s.hlju.edu.cn (H.F.); 2211697@s.hlju.edu.cn (Z.Z.); 2201611@s.hlju.edu.cn (J.Z.); 2211700@s.hlju.edu.cn (D.F.); 2211824@s.hlju.edu.cn (J.W.); 2211789@s.hlju.edu.cn (C.L.); 124wutong@163.com (T.W.); wang2687220886@163.com (G.W.); 20180813@s.hlju.edu.cn (L.W.); ranlingling@hlju.edu.cn (L.R.); 2 Heilongjiang Provincial Key Laboratory of Metamaterials Physics and Device, School of Electronic Engineering, Heilongjiang University, Harbin 150080, China

**Keywords:** terahertz, metamaterial, multi-functional, dual-broadband absorber

## Abstract

A dual broadband terahertz bifunction absorber that can be actively tuned is proposed. The optical properties of the absorber were simulated and numerically calculated using the finite-difference time-domain (FDTD) method. The results show that when the conductivity of vanadium dioxide is less than $\sigma_{0}=8.5\times 10^{3}$ S/m, the absorptance can be continuously adjusted between 2% and 100%. At vanadium dioxide conductivity greater than $\sigma_{0}=8.5\times 10^{3}$ S/m, the absorption bandwidth of the absorber can be switched from 3.4 THz and 3.06 THz to 2.83 THz and none, respectively, and the absorptance remains above 90%. This achieves perfect modulation of the absorptance and absorption bandwidth. The physical mechanism of dual-broadband absorptions and perfect absorption is elucidated by impedance matching theory and electric field distribution. In addition, it also has the advantage of being polarization insensitive and maintaining stable absorption at wide angles of oblique incidence. The absorber may have applications in emerging fields such as modulators, stealth and light-guided optical switches.

## 1. Introduction

In recent years, terahertz (THz) waves have attracted a great deal of attention because of their promising applications in wireless communications [1], sensors [2] and imaging [3]. As research into terahertz technology has advanced, various functional metamaterial devices have been proposed and theoretically investigated, such as fiber optic filters [4,5], absorbers [6,7,8,9,10], polarization converters [11] and hyperbolic metamaterials [12]. Among them, metamaterial perfect absorbers (MPAs) are widely used in the THz range due to their widespread applications in imaging [13,14], stealth technologies [15,16] and so on [17,18,19] and play a very important role within. In 2008, Landy et al. used a classical three-layer structure as a metamaterial perfect absorber (MPA) so that the incident wave was perfectly absorbed by the metamaterial absorber [20], and since then, various single-band [21,22,23,24], double-band [25,26,27,28] and multi-band absorbers [29,30] have been proposed. However, the absorption bandwidth of these MPAs are relatively narrow, and their electromagnetic responses cannot be adjusted after the structure has been determined, which limits their practical application. In order to achieve active tuning of the absorber in the terahertz band, we use the phase transition material vanadium dioxide (VO_2_) as the material for tuning the absorption. Many studies in recent years have shown that VO_2_ has significant optical change properties in the terahertz band and that the phase transition of VO_2_ from the insulator to the metal phase is around 340 K. It can be triggered by electrical [31,32,33], thermal [34,35] or optical excitation [36,37], with changes of several orders of magnitude in electrical conductivity during this time. Additionally, the phase transition from insulator to metal is accompanied by a steady increase in the conductivity of VO_2_. Therefore, utilizing vanadium dioxide as an absorber material is an effective way to realize the active tuning of absorbers [38,39,40,41].

In this work, a tunable dual-broadband terahertz absorber based on VO_2_ metamaterial is proposed. It consists of four vanadium dioxide fillet cavities, a TOPAS dielectric spacer layer, vanadium dioxide film, a silica dielectric layer and gold film from top to bottom. All materials are homogeneous [42]. The theoretical study of the absorber uses FDTD and impedance matching theory. The simulation results show that the efficiency and bandwidth of absorption can be changed by tuning the conductivity of vanadium dioxide. Moreover, the absorber is insensitive to the polarization of incident light and is able to maintain absorption stability at large angles of oblique incidence. The absorber provides a new scheme for tunable dual-broadband absorbers with broad application prospects in modulators, stealth and photoconductive light switches.

## 2. Materials and Methods

### Design and Simulation

**Figure 1:**
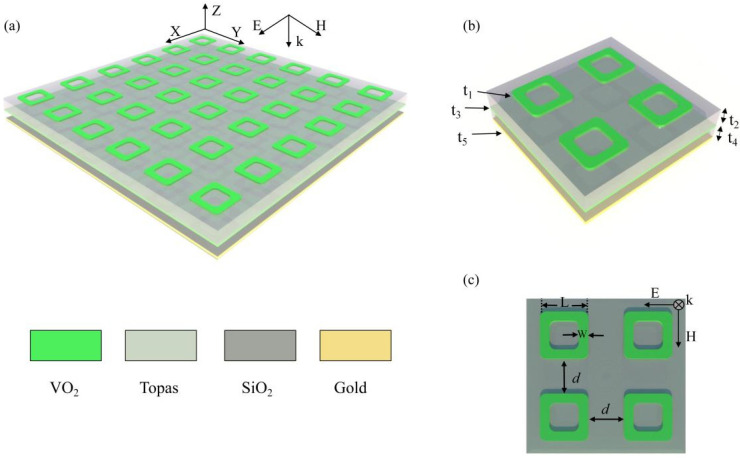
(**a**) Schematic diagram of the whole proposed 3D structure. (**b**) Schematic of a unit cell. (**c**) Top view of a unit cell.

As shown in Figure 1a,b, the schematic diagram of the designed metamaterial absorber, each cell is composed of four vanadium dioxide fillet cavities, a TOPAS dielectric spacer, a vanadium dioxide film, a silicon dioxide (SiO_2_) layer and gold film from top to bottom. The thicknesses of the vanadium dioxide fillet cavities, the TOPAS, vanadium dioxide thin-films, the silicon dioxide layer and the gold are t1 = 0.3 µm, t2 = 14 µm, t3 = 0.81 µm, t4 = 10 µm and t5 = 0.4 µm, respectively. Figure 1c is a top view of the unit, and the geometric parameters of the vanadium dioxide fillet cavity are L = 21 µm, d = 19 µm, w = 5.5 µm. The composite dielectric constant of the TOPAS is ε=2.35+0.01i. TOPAS is a transparent, easy to produce thermoplastic copolymer with constant refractive index and negligible absorption coefficient in the terahertz range, which is widely used in terahertz devices. The conductivity of gold and the relative permittivity of SiO_2_ are $\sigma =4.56\times 10^{7}$ S/m and ε=3.8, respectively. The performance of the designed absorber is theoretically studied by using the FDTD method (Lumerical Solutions, Vancouver, BC, Canada). In the simulations, the boundary conditions in the x and y directions are set as periodic boundaries and the boundary conditions in the z direction are set as perfectly matched layers. The absorption can be defined as $A\left(\omega \right)=1-R\left(\omega \right)-T\left(\omega \right)$, where $R\left(\omega \right)={\left|S_{11}\left(\omega \right)\right|}^{2}$ is the reflectance. As the thickness of the gold film is greater than the penetration depth of the THz wave, transmittance is $T\left(\omega \right)=0$. The optical permittivity of VO_2_ in the THz range can be described by the Drude model [43,44]:

(1) $$\varepsilon \left(\omega \right)=\varepsilon_{\infty }-\frac{\omega_{\rho }^{2}\left(\sigma \right)}{\left(\omega^{2}+i\gamma \omega \right)}$$

where $\varepsilon_{\infty }=12$ is the permittivity at the infinite frequency, $\gamma =5.75\times 10^{13}$ rad/s is the collision frequency and the plasma frequency at σ can be given by $\omega_{\rho }^{2}=\sigma \left(VO_{2}\right)\omega_{\rho }^{2}\left(\sigma_{0}\right)/\sigma_{0}$ with $\sigma_{0}=3\times 10^{5}$ S/m and $\omega_{\rho }\left(\sigma_{0}\right)=1.4\times 10^{15}$ S/m. In this paper, the conductivity of vanadium dioxide in the insulator phase and the metal phase are assumed to be $\sigma_{0}=0$ S/m and $\sigma_{0}=2\times 10^{5}$ S/m, respectively.

## 3. Results

**Figure 2:**
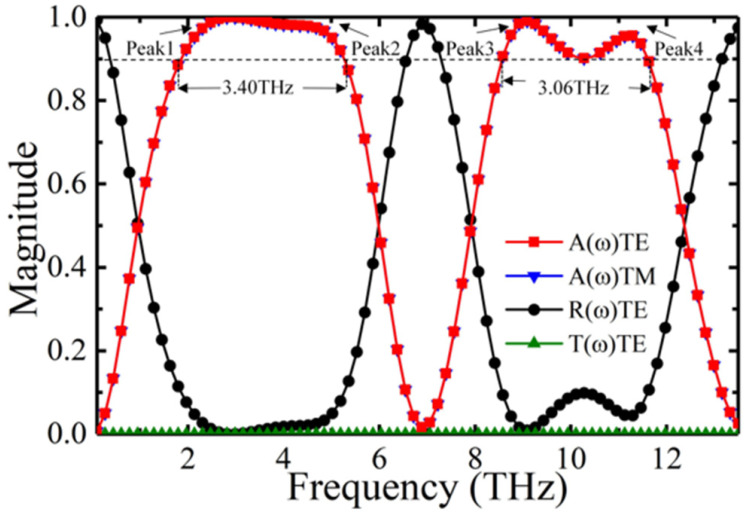
Absorption, reflection and transmission spectra.

Figure 2 shows the absorption, reflection and transmission spectra of the absorber at the vanadium dioxide conductivity $\sigma_{0}=8.5\times 10^{3}$ S/m. Due to the symmetry of the structure, the linear and elliptic angle polarization have no effect on absorption. In the linear angle polarization, the absorptance for the transverse electric (TE) and transverse magnetic (TM) polarizations are consistent. It follows that the absorber is polarization insensitive. The absorber exhibits outstanding absorptance and absorption bandwidth as the terahertz waves are confined to the surface by the vanadium dioxide, producing a localized surface plasma resonance. The absorption bandwidth of 90% absorptance under normal incidence is between 1.85 and 5.25 THz and 8.56 and 11.6 THz, up to 3.4 THz and 3.06 THz, respectively. There are four perfect absorption peaks, with Peak 1 = 2.95 THz, Peak 2 = 4.4 THz, Peak 3 = 9.1 THz and Peak 4 = 11.3 THz. Since there is a sufficiently thick gold film at the bottom of the absorber, the transmittance is 0. 

**Figure 3:**
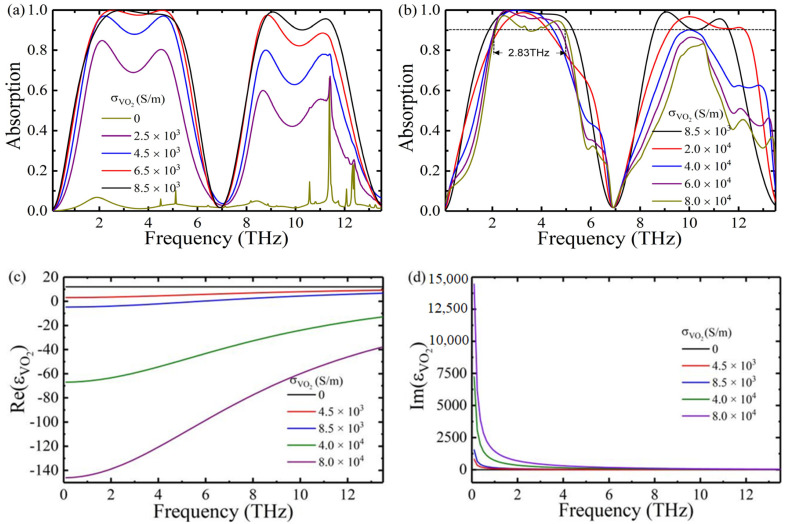
(**a**) Absorption spectrum of VO_2_ increasing from $\sigma_{0}=0$ S/m to $\sigma_{0}=8.5\times 10^{3}$ S/m and (**b**) $\sigma_{0}=8.5\times 10^{3}$ S/m to $\sigma_{0}=8.0\times 10^{4}$ S/m. (**c**) Real parts and (**d**) imaginary parts of permittivity with different conductivities of VO_2_.

We calculated the reflectance $R\left(\omega \right)={\left|S_{11}\left(\omega \right)\right|}^{2}$ and transmittance $T\left(\omega \right)=0$ of the absorber at different vanadium dioxide conductivities using the s-parameters. According to the formula for the absorption, $A\left(\omega \right)=1-R\left(\omega \right)-T\left(\omega \right)$, we can obtain the absorption spectrum of the absorber for different vanadium dioxide conductivities. The absorption spectrum of the conductivity of vanadium dioxide varying from $\sigma_{0}=0$ S/m to $\sigma_{0}=8.5\times 10^{3}$ S/m is shown in Figure 3a. As the conductivity increases, the absorptance can be dynamically adjusted from 2% to 100%. The main reason for this phenomenon is that it undergoes an optical transition from dielectric to metal when the conductivity increases from $\sigma_{0}=0$ S/m to $\sigma_{0}=8.5\times 10^{3}$ S/m. The greater the conductivity, the better the metal behavior and the higher the absorptance. As shown in Figure 3b, when the conductivity increases from $\sigma_{0}=8.5\times 10^{3}$ S/m to $\sigma_{0}=8.0\times 10^{4}$ S/m, the absorption bandwidth can be switched from 3.4 THz and 3.06 THz to 2.83 THz and none, respectively. The metal behavior of vanadium dioxide increases as its conductivity increases. Only the upper three-layer structure achieves absorption performance, and the lower layer structure does not work, so the absorption bandwidth decreases. The real and imaginary parts of the relative permittivity of the VO_2_ for different conductivities are shown in Figure 3c,d. The results show that the variation in the imaginary part is much greater than the variation in the real part for the different conductivities. This results in a significant variation in the absorptance and absorption bandwidth [25,43,44].

**Figure 4:**
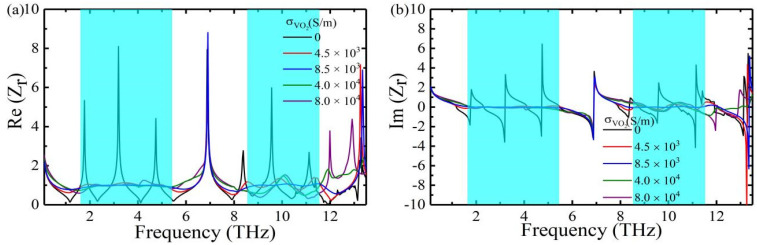
(**a**) Real parts and (**b**) imaginary parts of the relative impedance with different conductivities of VO_2_.

The absorption mechanism of this dual-broadband absorber can be well explained by impedance matching theory. When the effective impedance $Z_{r}=Z/Z_{0}=1$ of the absorber matches the effective impedance of the free space, the reflectance is minimized, and the absorptance and relative impedance can be obtained as follows:

(2) $$A\left(\omega \right)=1-R\left(\omega \right)=1-{\left|\frac{Z-Z_{0}}{Z+Z_{0}}\right|}^{2}=1-{\left|\frac{Z_{r}-1}{Z_{r}+1}\right|}^{2}$$

(3) $$Z_{r}=\pm \sqrt{\frac{\left(1+S_{11}\left(\omega \right)\right)-S_{21}{}^{2}\left(\omega \right)}{\left(1-S_{11}\left(\omega \right)\right)-S_{21}{}^{2}\left(\omega \right)}}$$

where $Z_{0}$ and Z are the effective impedance of the free space and the absorber, $Z_{r}=Z/Z_{0}$ is the relative impedance and $S_{11}\left(\omega \right)$ and $S_{21}\left(\omega \right)$ represent the reflectance and transmittance of the absorber, respectively. The gold film on the bottom of the absorber makes terahertz waves impenetrable, so $T\left(\omega \right)={\left|S_{21}\right|}^{2}$ is zero. Figure 4a,b shows the real and imaginary parts of the relative impedance of vanadium dioxide at different conductivities. The results show that when the conductivity of VO_2_ is $\sigma_{0}=8.5\times 10^{3}$ S/m, the real part gradually reaches 1 and the imaginary part gradually reaches 0 in the frequency ranges of 1.85–5.25 THz and 8.54–11.6 THz, respectively. It follows that the impedances of the absorber and free space gradually match, which can absorb the incident wave to the maximum extent.

**Figure 5:**
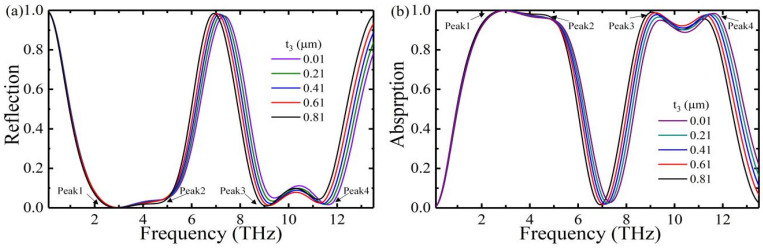
(**a**) Reflection spectrum and (**b**) Absorption spectrum of vanadium dioxide films of different thicknesses.

Furthermore, we also investigated the relationship between the reflectance and absorptance of this absorber and the thickness of the vanadium dioxide film. Figure 5a,b shows the reflectance and absorptance of vanadium dioxide at a fixed conductivity of $\sigma_{0}=8.5\times 10^{3}$ S/m. The results show that when the thickness of the vanadium dioxide film increases from 0.01 µm to 0.81 µm, the two broad absorption bands are red-shifted. As shown in Figure 5a, the reflectance of Peak 1 and Peak 3 gradually decreases, and the effective impedance of the absorber better matches the free space, resulting in increased absorptance of Peak 1 and Peak 3, as shown in Figure 5b. The reflectance of Peak 2 and Peak 4 are stronger and stronger, which causes a gradual mismatch between the effective impedance of the absorber and the free space, so the absorptance of Peak 2 and Peak 4 drops, as shown in Figure 5b. By comparing the reflectance and absorptance of the absorber with the thickness of the vanadium dioxide film, it is found that the absorber performs best at the thickness of 0.81 µm.

**Figure 6:**
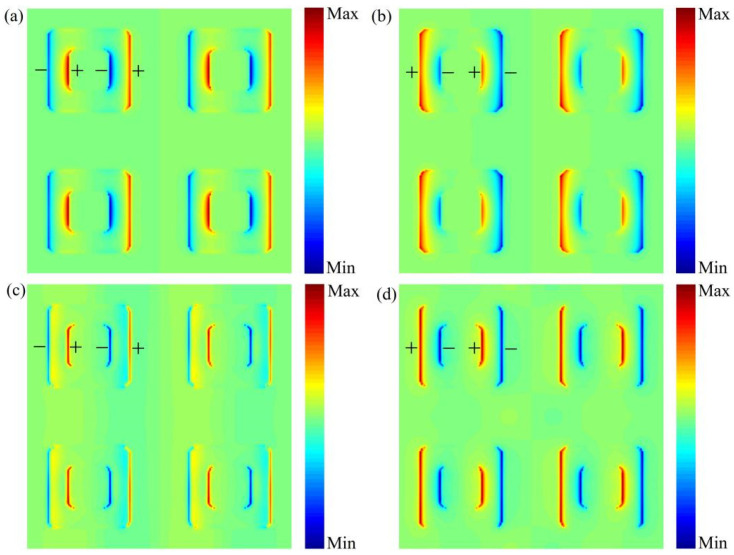
Electric field distribution at (**a**) Peak 1 = 2.95 THz. (**b**) Peak 2 = 4.4 THz. (**c**) Peak 3 = 9.1 THz. (**d**) Peak 4 = 11.3 THz.

To better explain the absorption mechanism of the dual-broadband absorption, we analyze the electric field distribution at the absorption peak. Figure 6a,c shows the electric field distributions at Peak 1 and Peak 3 when the vanadium dioxide conductivity is $\sigma_{0}=8.5\times 10^{3}$ S/m. The charge is distributed on the inner and outer sides of the vanadium dioxide in the form of “−+−+”, exhibiting the local plasmon resonance phenomenon. Similarly, as shown in Figure 6b,d, the electric fields at Peak 2 and Peak 4 exhibit local plasmon resonance, and the charges are distributed as “+−+−” on the inner and outer sides of the vanadium dioxide. It can be seen that the localized plasmon resonance is the main reason for the dual-broadband absorption of the absorber.

**Figure 7:**
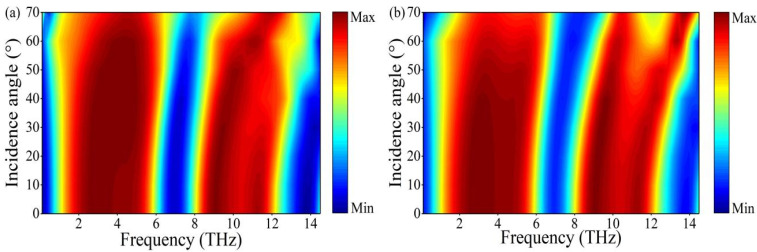
Absorption spectra of the absorber for (**a**) TE and (**b**) TM polarization at different oblique incidence angles.

The angle at which the absorber maintains stable absorption under oblique incidence is an important parameter to measure whether the absorber can be applied in practice. Therefore, we investigated the effect of the incident angles of THz waves on the absorptance. Here, we set the conductivity of the vanadium dioxide to $\sigma_{0}=8.5\times 10^{3}$ S/m and the thickness of the vanadium dioxide film to 0.81 µm. As shown in Figure 7a, the incident wave angle of TE polarization is increased from $0^{{}^{\circ}}$ to $70^{{}^{\circ}}$, the two broadband absorptions are slightly blue-shifted and a stable absorption bandwidth is maintained in the $0^{{}^{\circ}}$ to $60^{{}^{\circ}}$ range. The high-frequency absorption bandwidth becomes narrower when the incident angle is greater than $60^{{}^{\circ}}$. The variation of the two broadband absorptions with the TM polarization angle is shown in Figure 7b. As the angle of incidence increases, the two broadband absorptions gradually blue-shift. The broadband absorption at high frequencies splits into two absorption peaks after incident angles greater than $60^{{}^{\circ}}$, but the absorptance can still remain above 90%. From the above discussion, we can conclude that the structure is a tunable, polarization insensitive and a wide-angle dual-broadband perfect THz wave absorber.

In recent years, many new metamaterial absorbers have been proposed one after another. The comparisons of the absorption bandwidth, adjustable material, tunable function and oblique incidence of the absorber with those of similar articles are shown in Table 1. Our designed absorber can actively tune the absorptance and absorption bandwidth using vanadium dioxide. Additionally, the width of the absorption bandwidth and the oblique incidence angle for maintaining a stable absorptance are higher than those in previous reports, so this metamaterial perfect absorber has broad application prospects in modulators, stealth and light-guided optical switches.

## 4. Conclusions

In summary, we propose and study an actively tunable dual-broadband terahertz perfect absorber, each cell of which is composed of four vanadium dioxide fillet cavities, a TOPAS dielectric spacer, a vanadium dioxide film, a SiO_2_ layer and gold film from top to bottom. The absorption bandwidth can reach 3.4 THz and 3.06 THz with absorptance higher than 90% and is insensitive to the polarization angle. The absorptance and absorption bandwidth can be continuously adjusted by changing the conductivity of the vanadium dioxide. In addition, this design enables stable broadband absorption of TE and TM polarizations within oblique incidence angles of $70^{{}^{\circ}}$ and $60^{{}^{\circ}}$, respectively. The absorber may have a wide range of applications in THz devices such as modulators, cloaking devices and light-guided optical switches.

## Figures and Tables

**Table 1:** Comparison of this work with similar articles.

Reference	Absorption Bandwidth (THz)	Adjustable Material	Tunable Function	Oblique Incidence (TE)	Oblique Incidence (TM)
[17]	1.25	VO_2_	Absorptance	40°	60°
[19]	0.70	BDS, STO	Absorptance, frequency	40°	40°
[21]	0.88, 0.77	VO_2_	Absorptance	50°, 20°	60°, 20°
[22]	2.32, 2.03	VO_2_	Absorptance	Not given	Not given
This work	3.40, 3.06	VO_2_	Absorptance, bandwidth	70°, 60°	70°, 60°

## Data Availability

The data are included in the main text.
